# Making progress in a rare disease: emerging therapeutics in soft tissue sarcomas

**DOI:** 10.12688/f1000research.15868.1

**Published:** 2018-11-02

**Authors:** Jennifer Choe, Richard Riedel

**Affiliations:** 1 Duke University Medical Center, Durham, NC, USA

**Keywords:** Soft tissue sarcoma, novel therapies, immunotherapy

## Abstract

Sarcomas are rare tumors derived from mesenchymal connective tissues in the body. Because there are well over 50 histologic sarcoma subtypes, including malignant and non-malignant pathologies, clinical courses and therapeutic management are widely divergent. In general, therapeutic options across all soft tissue sarcomas are limited in number and are often generalized across multiple sarcoma histologies. The recent emergence of molecularly targeted therapies and immune-based agents presents a future of refined systemic treatment practices that are rationally tailored to the tumor by histologic subtype and biologic mechanisms.

## Introduction

Soft tissue sarcomas (STS) represent a heterogeneous group of rare mesenchymal neoplasms with varied clinical presentations and behavior. In 2018, 13,040 new cases will be diagnosed ^1^. Over 50 histologic subtypes have been identified, with the most commonly represented subtypes including gastrointestinal stromal tumors (GISTs), undifferentiated pleomorphic sarcomas, liposarcomas, and leiomyosarcomas. They can occur anywhere in the body and have widely variable clinical courses ranging from indolent to highly aggressive. In general, the most common sites of presentation are the extremities, but histologic subtypes demonstrate predilections for various parts of the body. The heterogeneity of histologic subtypes creates significant obstacles in identifying unifying treatment strategies across the many subtypes, since the rarity of these tumors has posed limitations in adequately powering clinical trials. The vast differences in biologic drivers of disease and clinical behavior have posed tremendous challenges to the development of effective treatment options for these tumors. Increasingly, clinical trials for STS have become more focused on specific histologic subtypes or tissue-agnostic molecular drivers of disease. The emergence of rationally designed studies and novel experimental therapeutics promises hope for expanded treatment options for patients with unresectable and metastatic STS. Here, we describe the most promising therapies to affect sarcoma management in the near future.

## Gastrointestinal stromal tumors

GISTs represent one of the most commonly diagnosed STS. Roughly 5,000 new cases are diagnosed in the United States annually. These tumors can occur anywhere along the GI tract and are thought to be derived from the interstitial cells of Cajal, the pacemakers of the gastrointestinal tract. GISTs are most commonly found in the stomach (60%), small intestine (30–35%), and other locations in the GI tract (<10%) ^2^. Gain-of-function mutations in the growth factor receptor genes for KIT (CD117) and platelet-derived growth factor α (PDGFRA) drive tumor development in 85–90% of GISTs to promote constitutive activation of growth factor signaling pathways that cause uncontrolled cell proliferation.

GISTs are considered unresponsive to traditional cytotoxic chemotherapies and radiation. Prior to 2000, no effective systemic therapeutic options existed for patients with unresectable or metastatic disease. In 2000, the first report of response to the tyrosine kinase inhibitor (TKI) imatinib mesylate was published, and this was soon followed by an open-label randomized multi-center clinical trial in 147 patients that demonstrated partial response in 53.7% and stable disease in 27.9% of patients ^3, 4^. Multiple subsequent trials have now firmly established imatinib as the first-line systemic therapy for the management of GISTs. Two other TKIs have since been approved by the US Food and Drug Administration (FDA) for the treatment of GISTs, including sunitinib and regorafenib. Both of these have limited response rates (14% for sunitinib and 4.5% for regorafenib) but did demonstrate improved progression-free survival (PFS) compared to placebo in phase III trials ^5, 6^. While imatinib re-introduction after progression on approved agents has demonstrated an improved PFS over placebo, the duration of benefit is transient (imatinib versus placebo 1.8 versus 0.9 months; *p*=0.009) ^7^. Other TKIs, such as pazopanib, sorafenib, and nilotinib, have demonstrated marginal activity but may be considered as “off-label” options when clinical trial options are unavailable ^8– 11^.

Mutational status of KIT and PDGFRA is crucial to predicting response rates and durability of response to imatinib therapy. The most common mutations found in untreated GISTs are in the juxtamembrane region, specifically in exon 11 (70%) and exon 9 (15%), with up to another 10% of primary mutations being found in PDGFRA ^12^. KIT exon 11 mutations and insertions portend favorable prognosis, while deletions in exon 11 and exon 9 portend decreased durability of response and shorter survival rates ^13^. The most common PDGFRA mutation is the D842V substitution in the activation loop of PDGFRA. Tumors harboring this mutation are refractory to nearly all available treatment options and are associated with poor outcomes with a short PFS of only 2.8 months with imatinib ^14^.

The major challenge facing the treatment of GISTs remains resistance to currently available targeted therapies. Imatinib functions by binding the ATP-binding pocket for KIT and PDGFRA to prevent the activation of downstream signaling. Development of mutations in the ATP-binding pocket or activation loop decreases the drug’s binding affinity, leading to TKI resistance. The most commonly found secondary resistance mutations are encoded in exons 13 and 14 of the ATP-binding pocket and in exons 17 and 18 of the activation loop. About 14% of tumors have primary resistance to imatinib (progression within the first 6 months of treatment), while in 40–50% of tumors secondary resistance due to acquired mutations in KIT or PDGFRA develops after about 2 years on imatinib treatment ^3, 12^. Activation loop mutations accumulate with sunitinib treatment. For sunitinib and regorafenib, resistance typically develops within approximately 6 months of therapy ^5, 6^. Selection of agents based on the initiating and acquired resistance mutations in KIT and PDGFRA will hopefully expand systemic options beyond the three currently approved TKIs.

Currently, no approved treatment options are available for the highly resistant PDGFRA D842V exon 18 activation loop mutation. However, a number of promising therapeutic agents have emerged. Crenolanib has been specifically developed to target this mutation and demonstrated a clinical benefit rate of 31% in 16 patients ^15^. A randomized phase III study is currently underway evaluating the efficacy of crenolanib compared to placebo in patients with PDGFRA D842V tumors (CrenoGIST: NCT02847429) ^16^.

BLU-285, also known as avapritinib, and DCC-2618 are highly potent and selective inhibitors of mutant KIT and PDGFRA, with demonstrated activity against PDGFRA D842V mutations along with secondary KIT resistance mutations. BLU-285 is a selective inhibitor of KIT and PDGFRA activation loop mutants ^17^. While BLU-285 demonstrated strong activity against clinically relevant single mutations in either the activation loop or the ATP-binding pocket of KIT and PDGFRA, tumor sensitivity was increased in the setting of dual mutants of the juxtamembrane and ATP-binding pocket and protein regions (e.g. exon 11/exon 17). In a phase I study of BLU-285, all 31 patients with D842V PDGFRA GISTs demonstrated a tumor response ^18^. A phase III study of BLU-285 compared to regorafenib in the third-line setting is planned (VOYAGER: NCT03465722).

DCC-2618 is a pan-KIT and PDGFRA inhibitor with activity against both initiating and acquired resistance mutations (KIT exons 9, 11, 13, 14, 17, and 18 and PDGFRA exon 18). Updated results from a phase I study of 57 heavily pre-treated GIST patients with two or more prior agents were presented at the European Society of Medical Oncology (ESMO) 2017 meeting, revealing a disease control rate of 76% at 12 weeks. DCC-2618 showed partial metabolic response in 69% of patients with KIT or PDGFRA mutants ^19^. Based on these encouraging results, a randomized phase III pivotal study in GIST patients for treatment in the fourth-line setting is ongoing (INVICTUS: NCT03353753). A second randomized phase III study in the second line versus sunitinib is planned.

## Non-gastrointestinal stromal tumor soft tissue sarcomas

### Unselected soft tissue sarcomas

With the exception of GISTs, clinical studies examining treatment for sarcomas historically included unselected patient populations. Treatment efficacy for these studies typically combined data for all histologic subtypes given the challenges in recruiting for adequately powered phase III studies for selected subpopulations. As a result, systemic options have traditionally been generalized to all STS despite limited patients enrolled for an individual subtype.

The mainstay of STS management is cytotoxic chemotherapy. Most commonly, the first-line treatment is an anthracycline-based regimen. Other combinations and cytotoxic agents commonly used have traditionally included gemcitabine-based regimens, single-agent dacarbazine, and single-agent ifosfamide. The multi-targeted TKI pazopanib was FDA approved for STS in 2012 ^20^. In general, however, response rates with cytotoxic agents have been relatively low, with short PFS intervals and associated toxicity.

Despite the obstacles faced in a “one-size-fits-all” treatment mentality, some recent treatments have emerged for unselected sarcomas. In a randomized, double-blind, phase II study, the multikinase inhibitor regorafenib demonstrated improvements in PFS in some anthracycline-refractory STS ^21^. Selected subtype clinical trials with regorafenib are currently ongoing (NCT02048371 and NCT02048722). The doxorubicin pro-drug aldoxorubicin demonstrated improved PFS compared to doxorubicin in a multicenter, randomized, phase IIb trial ^22^. In addition, a significantly improved cardiotoxicity profile was observed ^23^. The most pivotal advance in the treatment of STS was observed in a phase I/II study examining the combination of the PDGFRA antibody olaratumab with doxorubicin ^24^. While the study met its pre-defined primary endpoint, with an improvement in PFS of 2.5 months, it demonstrated a striking improvement in median overall survival (OS) of 11.8 months (26.5 months versus 14.7 months, *p*=0.0003) for the combination compared to doxorubicin monotherapy, resulting in FDA approval. A phase III confirmatory study with the combination is fully enrolled, and the results are eagerly awaited (ANNOUNCE: NCT02451943). Ongoing studies are exploring olaratumab in combination with gemcitabine/docetaxel (ANNOUNCE2: NCT02659020) as well as doxorubicin/ifosfamide (NCT03283696).

### Selected soft tissue sarcomas

The recognition for therapy tailored to histologic subtype and molecular drivers has led to a shift in the approach to clinical trial design for STS. This is demonstrated by the higher sensitivities of certain histologies to specific chemotherapies, such as paclitaxel for angiosarcoma or ifosfamide for synovial sarcoma ^25, 26^.

Liposarcomas and leiomyosarcomas, or so-called “L-type” sarcomas, comprise nearly half of unresectable and metastatic adult STS and therefore represent the largest STS population in need of therapeutic options. Liposarcomas, with the potential exception of myxoid liposarcomas, are considered relatively insensitive to chemotherapy. Eribulin and trabectedin are recently approved agents that have demonstrated modest activity in the L-type sarcomas. In a phase III study, patients with liposarcomas and leiomyosarcomas were randomized to receive either the microtubule polymerization inhibitor eribulin or dacarbazine ^27^. While eribulin improved OS by 2 months in comparison to dacarbazine (13.5 versus 11.5 months, hazard ratio [HR] 0.77, 95% confidence interval [CI] 0.62–0.95, *p*=0.0169) for all subjects enrolled, the benefit was seen primarily in the liposarcoma cohort. Based on these results, FDA approval for eribulin was granted in 2016 for use in liposarcomas but not for leiomyosarcomas. A recently published histology-specific subgroup analysis of liposarcomas revealed an improvement in OS of 15.6 months versus 8.4 months (HR 0.51, 95% CI 0.35–0.75, *p*<0.001) in addition to a PFS benefit of 2.9 versus 1.7 months (HR 0.52, 95% CI 0.35–0.78, *p*=0.0015) ^28^.

The DNA damage modulator trabectedin gained FDA approval for liposarcomas and leiomyosarcomas in 2015. While the phase III study failed to meet its primary endpoint of improved OS, trabectedin was approved based on a PFS benefit of 4.2 months compared to 1.5 months for dacarbazine (HR 0.55, 95% CI 0.44–0.70, *p*<0.001) ^29^. In published phase II and phase III studies, trabectedin appeared to be more effective in myxoid liposarcomas ^29, 30^.

The SINE (XPO-1) inhibitor selinexor is being explored in ongoing studies in dedifferentiated liposarcoma. Phase Ib results published in 2016 demonstrated stable disease in 7 of 15 (47%) patients for ≥4 months ^31^. An ongoing randomized, multi-center, double-blind, placebo-controlled phase II/III study is evaluating selinexor versus placebo in 245 patients with dedifferentiated liposarcoma. Results from the phase II portion of the study were presented at the 2018 American Society of Clinical Oncology (ASCO) meeting and revealed an improved PFS of 5.6 months versus 1.8 months by RECIST v1.1 criteria ^32^.

In a phase II study, the multi-targeted TKI anlotinib demonstrated activity in leiomyosarcoma and synovial sarcoma and appeared particularly promising in alveolar soft part sarcoma (ASPS) ^33^. A phase III study with anlotinib in these three subtypes is actively recruiting (APROMISS: NCT03016819). In results presented at ASCO 2018, patients with the typically chemotherapy-insensitive ASPS demonstrated an improvement in median PFS of 18.23 months compared to 3 months for placebo (HR 0.14, 95% CI 1.19–4.81, *p*<0.0001) in 56 ASPS patients ^34^.

Angiosarcomas are tumors derived from endothelial cells. Given the role of the vascular endothelial growth factor (VEGF) family of angiogenic factors, TKI therapies with anti-VEGF activity have been tested in sarcoma with variable results. Pazopanib has demonstrated similar efficacy in angiosarcomas as compared to other STS ^20, 35^. Other TKIs, such as sorafenib, have shown the ability to provide stable disease ^36^. The benefit of the anti-VEGF monoclonal antibody bevacizumab in the treatment of unselected STS, including angiosarcomas, appears limited ^37– 39^.

TRC105 is a novel antibody directed against endoglin, a molecule upregulated as a possible mechanism of resistance in response to VEGF inhibition and particularly densely expressed in angiosarcomas ^40, 41^. In a phase Ib/II trial, a small cohort of 18 angiosarcoma patients treated with TRC105 in combination with pazopanib or as a single agent followed by the addition of pazopanib at progression revealed a PFS of 5.59 months for the combination therapy, including two complete responses ^42^. A randomized phase III study of TRC105 in combination with pazopanib versus pazopanib alone in cutaneous and non-cutaneous angiosarcoma is ongoing (TAPPAS: NCT02979899).

## Locally aggressive non-malignant soft tissue tumors

Although not classified as malignant given the lack of metastatic potential, certain connective soft tissue tumors cause significant morbidity owing to locally infiltrative disease and mass effect with a high propensity for local recurrence. These include desmoid tumors and tenosynovial giant cell tumors (TGCTs)/pigmented villonodular synovitis (PVNS).

Historically, the management of desmoid tumors included surgical resection, but, given the morbidity and high local recurrence rates, conservative measures, in particular observation, are now increasingly favored. In symptomatic desmoid tumors, new therapies have arisen that target proliferative mechanisms of tumor growth. No FDA-approved therapies exist for desmoid tumors, and the selection of systemic therapy is based, in part, on patient preference and co-morbidities. Efficacy data are limited to small retrospective studies for traditional agents, including non-steroidal anti-inflammatory drugs (NSAIDs), anti-hormonal drugs, and cytotoxic agents such as liposomal doxorubicin. Because of increased expression of c-KIT and PDGFRA/B, TKIs such as imatinib and sorafenib have been investigated as potential therapies. Phase II studies with imatinib demonstrated low response rates from 5–19% with a 6-month PFS of 63% ^43– 45^. Slightly improving upon this, a retrospective study of 26 patients receiving sorafenib showed an objective response in 25% and symptomatic clinical benefit in 70% of patients ^46^. This prompted a randomized, double-blind phase III study of sorafenib versus placebo in 87 patients. At interim analysis, this study showed that sorafenib showed a benefit of PFS, which was not reached, compared to 11.3 months for placebo (HR 0.14, 95% CI 0.06–0.33, *p*<0.0001) and an objective response rate of 33% for sorafenib versus 20% in the placebo group (Alliance A091105: NCT02066181) ^47^. A 20% response rate was observed for patients who received placebo, reinforcing observation as an option for these patients given the potential for spontaneous regression.

In TGCTs/PVNS, a translocation involving the colony-stimulating factor (CSF1) gene promotes proliferative inflammation of the synovium through the recruitment of tumor-associated macrophage cells expressing the CSF1 receptor (CSF1R) ^48, 49^. Surgical resection is the mainstay of treatment but has the potential for significant morbidity and high risk for local recurrence. Previously, TGCTs/PVNS had no effective medical therapies. TKIs with activity against the CSF1/CSF1R pathway, such as imatinib and nilotinib, have previously been evaluated with limited objective responses, although disease stabilization was seen in a majority of patients ^50, 51^. The TKI pexidartinib (PLX3397) blocks the CSF1/CSF1R axis, which inhibits tumor-associated macrophage infiltration along with autocrine and paracrine signaling of CSF1 to inhibit tumor cell growth ^52^. A marked objective response and durable response in some patients was seen in early studies resulting in the development of the phase III ENLIVEN study (NCT02371369) ^53, 54^. In this study, 120 patients were randomized to either pexidartinib or placebo. A 39% overall response rate (ORR) at week 25 with no further progression at the median 6-month follow-up was seen in responders, which was a vast improvement over prior systemic therapies. Improvements were seen also in a number of functional and symptom scales. In addition, emactuzumab, a monoclonal antibody targeting CSF1R, showed an 86% objective response rate in a phase I study of TGCT/PVNS patients ^55^.

## Immunotherapy

With the excitement of immune checkpoint blockade in many tumor types, a number of trials have evaluated immunotherapy in the setting of metastatic STS. In general, responses have been limited across a broad range of histologic subtypes. However, selected subtypes of STS appear to demonstrate potential activity. These include undifferentiated pleomorphic sarcomas and dedifferentiated liposarcomas with objective response rates of 40% and 20%, respectively (n=10 for each histologic subtype), with pembrolizumab monotherapy in the SARC028 study ^56^. In the ALLIANCE study of nivolumab with or without ipilimumab, nivolumab monotherapy demonstrated a response rate of 5%, while the combination of nivolumab with ipilimumab revealed a response rate of 16% ^57^. Combination immune checkpoint strategies continue to be evaluated in multiple histology-specific phase I/II studies. In data presented at the 2018 ASCO annual meeting, pembrolizumab combined with axitinib, a small molecule inhibitor targeting VEGFR, c-KIT, and PDGFR, demonstrated promising activity in STS, particularly in ASPS, where an ORR of 45% was reported with preliminary evidence of durable responses ^58^. Case reports and small case series have additionally supported the concept of checkpoint inhibition in ASPS ^59, 60^.

Novel strategies of targeting tumor antigens, such as New York esophageal squamous cell carcinoma 1 (NY-ESO-1), are under active investigation and include adoptive T-cell transfer, chimeric antigen receptor-T cell, and vaccine-based therapies. These are being explored particularly in synovial and myxoid/round cell liposarcoma where >80% of tumors express the antigen (NCT03520959) ^61– 63^.

## Neurotrophic tyrosine receptor kinase-targeted therapies

The recent emergence of agents targeting neurotrophic tyrosine receptor kinase (NTRK) has generated significant excitement given case reports of marked responses in NTRK-rearranged sarcomas ^64^. These molecularly targeted agents are currently under study in tissue-agnostic phase II basket studies. In the NAVIGATE study, larotrectinib (LOXO-101) demonstrated an 80% ORR in all tumor types, including in 10 of 11 non-GIST STS and 3 of 3 GISTs ^64^.

Entrectinib (RDX-101), an oral inhibitor with activity against NTRK, ROS1, and ALK-rearranged tumors, has shown promising activity in early phase clinical trials and is being studied in the phase II STARTRK-2 study (NCT02568267) ^65^.

## Summary of emerging therapeutics in sarcoma

As we enter a new era of precision medicine and immunotherapy, the future of sarcoma appears poised for the discovery of rationally targeted and histology-specific therapeutic agents. The major obstacles facing sarcoma management remain the rarity and heterogeneity of the disease as well as the potential challenges of recruiting enough patients to a subtype-specific trial to make reasonable conclusions about therapeutic efficacy. With these challenges, developmental therapeutics in sarcoma have smartly shifted to targeting the tumors based on biologic mechanisms. A number of therapeutic agents are poised to change the management for some histologic subtypes ( Table 1). Increased understanding of the molecular pathogenesis of histologic subtypes has widened the therapeutic possibilities. The future will entail taking advantage of the unique biology of each histologic sarcoma subtype and identifying relevant biomarkers to drive therapeutic decision making in order to improve outcomes for this unique patient population.

**Table 1.** Ongoing late-phase clinical trials in sarcomas.

Sarcoma subtype	Agent(s)	Therapeutic target	Phase	Trial number
**GIST**	BLU-285	PDGFRA D842V Dual mutation (KIT exon 11/17)	Phase III	NCT03465722
**GIST**	DCC-2618	KIT exons 9, 11, 13, 14, 17, and 18; PDGFRA exon 18, including D842V	Phase III	TBD: NCT03353753
**GIST**	Crenolanib	PDGFRA D842V	Phase III	NCT02847429
**Desmoid tumor**	Sorafenib	PDGFR, VEGFR	Phase III	NCT02066181
**Unselected soft tissue** **sarcoma**	Doxorubicin/olaratumab	PDGFRA	Phase III	NCT02451943
**Dedifferentiated liposarcoma**	Selinexor	XPO-1	Phase II/III	NCT02606461
**Alveolar soft part sarcoma/** **synovial sarcoma/** **leiomyosarcoma**	Anlotinib	Multi-target (e.g. VEGFR2/3, PDGFRA/B, KIT)	Phase III	NCT03016819
**Synovial sarcoma**	CMB305	NY-ESO-1 vaccine		NCT03520959
**Angiosarcoma**	TRC105 + pazopanib	Endoglin and VEGFR1/2/3	Phase III	NCT02979899
**Tenosynovial giant** **cell tumor/pigmented** **villonodular synovitis**	Pexidartinib	CSF1R	Phase III	NCT02371369

CSF1R, colony-stimulating factor 1 receptor; GIST, gastrointestinal stromal tumor; NY-ESO1, New York esophageal squamous cell carcinoma 1; PDGFRA, platelet-derived growth factor receptor α; VEGFR, vascular endothelial growth factor receptor

## Abbreviations

ASCO, American Society of Clinical Oncology; ASPS, alveolar soft part sarcoma; CI, confidence interval; CSF1, colony-stimulating factor 1; CSF1R, colony-stimulating factor 1 receptor; FDA, US Food and Drug Administration; GIST, gastrointestinal stromal tumor; HR, hazard ratio; NTRK, neurotrophic tyrosine receptor kinase; ORR, overall response rate; OS, overall survival; PVNS, pigmented villonodular synovitis; PDGFRA, platelet-derived growth factor receptor α; PFS, progression-free survival; STS, soft tissue sarcoma; TGCT, tenosynovial giant cell tumor; TKI, tyrosine kinase inhibitor; VEGF, vascular endothelial growth factor; VEGFR, vascular endothelial growth factor receptor
